# In silico design and validation of a highly degenerate primer pair: a systematic approach

**DOI:** 10.1186/s43141-020-00086-y

**Published:** 2020-11-17

**Authors:** Prosper Obed Chukwuemeka, Haruna Isiyaku Umar, Oluwatoyin Folake Olukunle, Oluwaseyi Matthew Oretade, Christopher Busayo Olowosoke, Emmanuel Oluwasegun Akinsola, Michael Omoniyi Elabiyi, Usman Garba Kurmi, Joy Oseme Eigbe, Bukola Rukayat Oyelere, Lucky Efe Isunu, Oyeyemi Janet Oretade

**Affiliations:** 1 Department of Biotechnology, School of Sciences (SOS), Federal University of Technology Akure, Akure, P.M.B 704 Nigeria; 2 Department of Biochemistry, School of Sciences (SOS), Federal University of Technology Akure, Akure, P.M.B 704 Nigeria; 3 Department of Mechanical Engineering, School of Engineering and Engineering Technology (SEET), Federal University of Technology Akure, Akure, P.M.B 704 Nigeria; 4 Department of Microbiology, School of Sciences (SOS), Federal University of Technology Akure, Akure, P.M.B 704 Nigeria; 5 Department of Biochemistry, University of Maiduguri, Maiduguri, Nigeria; 6 Department of Biomedical Technology, School of Health and Health Technology (SHHT), Federal University of Technology Akure, Akure, P.M.B 704 Nigeria; 7 Department of Physiology, College of Health Science (CHS), Osun State University, Osogbo, Nigeria

**Keywords:** Accessible, Bioinformatics, Coverage, C12O, Degenerate primers, Genetic materials, HYDEN, In silico PCR, MC-DPD, Systematic

## Abstract

**Background:**

The techniques of amplifying genetic materials have enabled the extensive study of several biological activities outside the biological milieu of living systems. More recently, this approach has been extended to amplify population of genes, from evolutionarily related gene family for detection and evaluation of microbial consortial with several unique potentialities (e.g., enzymatic degradability). Conceivably, primer mixtures containing substitutions of different bases at specific sites (degenerate primers) have enabled the amplification of these genes in PCR reaction. However, the degenerate primer design problem (DPD) is a constraint to designing this kind of primer. To date, different algorithms now exist to solve various versions of DPD problem, many of which, only few addresses and satisfy the criteria to design primers that can extensively cover high through-put sequences while striking the balance between specificity and efficiency. The highly degenerate primer (HYDEN) design software program primarily addresses this variant of DPD problem termed “maximum coverage-degenerate primer design (MC-DPD)” and its heuristics have been substantiated for optimal efficiency from significant successes in PCR. In spite of the premium presented for designing degenerate primers, literature search has indicated relatively little use of its heuristics. This has been thought to result from the complexity of the program since it is run only by command-line, hence limiting its accessibility. To solve this problem, researchers have optionally considered the manual design of degenerate primers or design through software programs that provides accessibility through a graphical user interface (GUI). Realizing this, we have attempted in this study to provide a user-friendly approach for researchers with little or no background in bioinformatics to design degenerate primers using HYDEN

**Results:**

Virtual Tests of our designed degenerate primer pair through in silico PCR substantiated the correspondence between efficiency and coverage with the target sequences as pre-defined by the initial HYDEN output, thereby validating the potentials of HYDEN to effectively solve the MC-DPD problem. Additionally, the designed primer-pair mechanistically amplified all sequences used as a positive control with no amplification observed in the negative controls.

**Conclusion:**

In this study, we provided a turnkey protocol to simplify the design of degenerate primers using the heuristics of the HYDEN software program.

## Background

The advent of polymerase chain reaction (PCR) has revolutionize the understanding of genetic materials (DNA and RNA) with a wide range applicability in many biological studies including amplification, gene expression, cloning, mutation detection, mutagenesis, and a large list of genome typing experiments that are of pertinence in the metagenomics era. PCR is not a technique limited to just a single field of study and has been exploited in several interdisciplinary researches spanning across specialties such as molecular biology, medicine, biotechnology, agriculture, engineering, biochemistry, microbiology, genetics, and a good number of fascinating applications in newer scope of biological or life sciences. Polymerase chain reaction is an in vitro technique used to make genetic materials (DNA) in several orders of magnitude by amplifying DNA segment of known sequences or a portion of DNA that lies between two known sequences to exponential copies. The technique generally involves three steps all of which are temperature-dependent: denaturation, annealing (primer hybridization), and extension (primer elongation) [1].

To execute PCR, the genetic material is first denatured in a melting step involving thermal elevation of double-stranded DNA molecules to a temperature nearly at the boiling point of ordinary water, thus, converting double-stranded DNA molecules within a reaction mixture into single strands. Subsequently, short single-stranded nucleotide strings called “primers” bind to complementary regions within the melted DNA molecule in a process called hybridization. Accordingly, the resulting single -stranded DNA templates are enzymatically extended by the activity of a thermostable polymerase (called DNA polymerase) into new double-stranded DNA from free nucleotides contained within a reaction mixture. Verbally, the overall workflow of a polymerase chain reaction (PCR) can be described as a simple biological process. However, ascertaining the success of PCR is more cumbersome than it looks since the technique is very sensitive and highly susceptible to contamination which may result in false positivity [2]. To make PCR a specific, efficient, and cost-effective tool for researchers and scientists, nucleic acid template of sufficiently high quality which should be free of DNA polymerase inhibitors and the selection of appropriate oligonucleotide primers are of pertinence for the overall success of the experiment [2, 3]. Since DNA polymerases do not possess de novo activity and often requires a free 3’ –OH end to facilitate DNA elongation, the role of primers in PCR studies is of great consideration and cannot be overruled. Owing to this striking point, various paradigms to design primers have been proposed over time and several commercial software programs have been developed to design different types of primers that would fit the primer design constraints. Correspondingly, free web servers to effectively determine the properties of these oligonucleotide strings have also been made available thus, reducing time implications, ambiguity, and errors of humans that might occur from the manual design of primers, hence resulting in improved primer design accuracy [4].

To date, different types of primers now exist with each specific to the experiment under consideration. PCR primers may be designed to suit a wide range of studies including amplification, gene expression, cloning, mutation detection, mutagenesis, molecular fingerprinting, among others. Though some primers are generated by single or the synergy of two or more programs and may require different computational algorithm depending on the heuristics of the program been used, nonetheless, they all explore a set of common criteria (e.g., % GC content, melting temperature, primer length, etc.) to evaluate the quality of designed candidates in the specified region within the target selected by the user [5].

In the context of genome evolution, evolutionary divergence, and species formation, evolutionary dictates encrypting amino acid sequences are highly conserved [6]. While certain amino acids of a protein are conserved among species, the corresponding codons may differ due to degeneracy (ability to choose from four nucleotide bases). In spite of this redundancy in the codons, primer mixtures that have substitutions of different bases at specific sites will enable the amplification of closely related gene homologs in a PCR reaction and has been widely explored. PCR primers designed for this purpose are termed “degenerate primers” and has been more recently used in several molecular studies to detect and evaluate genes within microbial populations that are capable of several unique potentialities with the advent of various DPD (degenerate primer design) paradigms. In spite of the knowledge from this approach, literature search has indicated insufficient studies on DPD compared to other primer types. To date, more written programs developed to solve emerging consensus regarding primer design and optimal primer properties are non-degenerate based with little programs readily available to handle these constraints in degenerated primers. Since their design is rather classified as an optimization problem [7]. Relatively few heuristic algorithms have been useful in addressing these challenges. In an earlier day, Rose et al. [8] proposed Consensus-Degenerate Hybrid Oligonucleotide Primer (CODEHOP) which is capable of finding primers in the conserve region of amino acid sequences. Additionally, Wei et al. [9] also designed degenerate primers from aligned protein sequences to identify new members of protein families using their DePiCt model. Accordingly, Meyer et al. aligned unknown DNA sequences with known DNA sequences, that can perform similarly functions, from which primer capable of dealing with the unknown DNA sequences were designed [4]. Other available DPD programs also include GeneFisher [10], DPP [6], Greene SCPrimer [11], DegePrime [12], FAS-DPD [5], etc. Although a significant level of success has been reported with many of these DPD tools in actual PCR experiment, nevertheless, a more disturbing constraint to use may arise from DPD problem. This describes the algorithmic search of primers that included degenerated positions [5]. Although the DPD problem has been described in various contexts by several researchers [12–14]. Generally, only two established variants of this problem have been extensively emphasized (the minimum degeneracy DPD and maximum coverage DPD) and have been the main focus to optimize older and newer DPD programs. The initial attempts to find primers with minimum degeneracy that covers all the input strings [13], while the latter, tries to find a minimum number of primers that together matches all the input sequences with a level of degeneracy below the set threshold.

In the context of MC-DPD, the open source highly degenerate (HYDEN) primer program by Linhart and Shamir [7] basically addresses this form DPD problem. Their model presents an ultimate premium for designing degenerated oligonucleotides from closely related homologs and could be used with larger sets of sequences unlike other paradigm such as CODEHOP. In spite of the extent of HYDEN’s computational algorithm to optimize for promising degenerate primer sets, its use for selecting primers is depreciated by the lack of a GUI (graphical user interphase) suggesting the need for some level of expertise prior to use. Realizing the potentials of HYDEN as a powerful DPD tool and the need for designing more novel degenerate primers with greater efficiency, and higher specificity that could be employed for use in many biological researches, we have attempted in this study to provide an explicit approach for designing degenerate PCR primers via the heuristics of HYDEN software program, and we have anticipated making the program easier for use by researchers and scientists with little or no background in computer-aided primer design. For the purpose of this study, we have designed a highly degenerate primer pair targeting a set of catechol 1,2-dioxygenase (C12O) genes among 88 bacterial strains.

## Materials

It is noteworthy that the design of the degenerate primer pair reported in this present study was effectively executed by the synergy of different software programs and web servers. The software programs used here include the open-sourced Highly Degenerate primer (HYDEN) design program accessible from (http://acgt.cs.tau.ac.il/hyden/hyden_license.html) [7], FastPCR v6.7 (http://primerdigital.com/Fastpcr.html) [14], Geneious Prime software version2020.1.2 (www.geneious.com/prime/). The degenerate primer pair reported in this study was designed on a hp personal computer composed of a 64-bit operating system, ×64-based processor, 2 CPUs, and a storage of 500 GB. The material used in this study were 88 catA genes from authentic bacterial strains known to possess the catabolic gene. The gene sequences were downloaded in FASTA format from NCBI database accessible from (https://ncbi.nlm.nih.gov). Files interconversion from the extension .txt to FASTA format was achieved through an open-sourced web server accessible from (http://www.hiv.lanl.gov/content/sequence/FORMAT_CONVERSION/form.html).

## Methods

### Retrieval of sequence and library construction

**Fig. 1:**
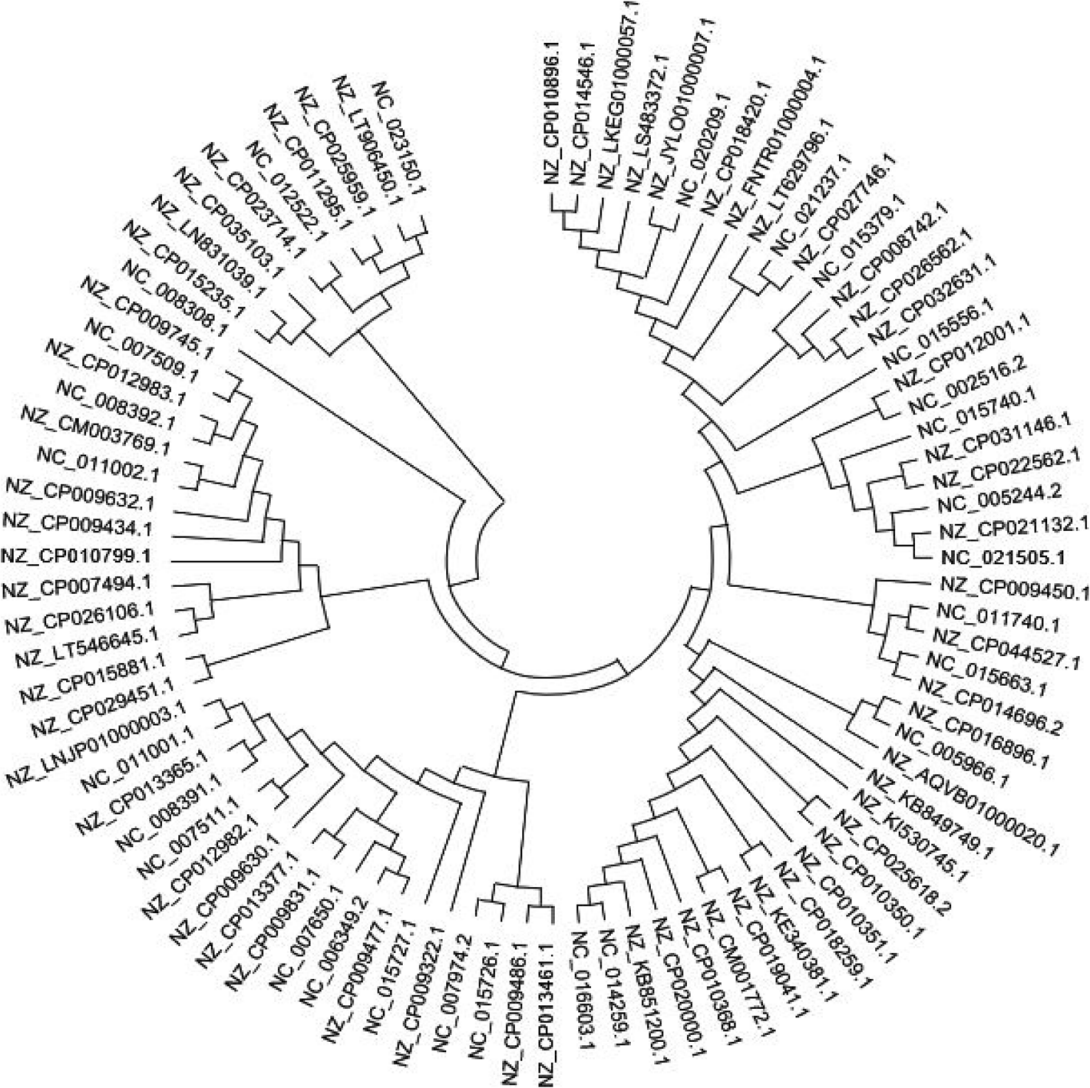
Phylogenetic tree showing the relationship among catA sequences obtained from NCBI. The tree was constructed by neighbor-joining method. Genebank accession numbers are given at the terminal of the tree (leaf)

The gene encoding the catabolic enzyme C12O (i.e, catA), an intradiol dioxygenase primarily involved in aromatic compound mineralization is widely dispersed among several bacterial populations, more of which the phylum proteobacteria holds a good profile of this gene. C12O mechanistically remediate several recalcitrant aromatic compounds from a wide variety of environment via aerobic degradation through the catechol ring-cleavage pathway and has been observed among other bacteria phylum including actinobacteria. For the purpose of this study, nucleotide search was conducted for the catabolic gene (catA) against the curated NCBI primary database (https://ncbi.nlm.nih.gov) from which a total of 88 Cl20 gene sequences (all of 1 kb in size in the coding region of C12O genes) were downloaded in FASTA format. All the sequences obtained were selected from authenticated bacterial strains known to have the catabolic gene (catA) in the phylum proteobacteria and actinobacteria respectively (Table 1). The sequences were organized using a text editor and saved as a single txt file, which was thereafter converted into FASTA format [7]. The list of bacterial strains used to design the degenerate primer pair is listed in (Tables 6, 7, 8, 9, 10, and 11). (Figure 1) depicts the evolutionary relatedness of all bacterial strains used in the design of the degenerate primer reported in this study.

**Table 1:** Taxonomic characterization of bacterial strains used for degenerate primer design

Phylum	Class	Number of retrieved sequences from database
Proteobacteria	α-proteobacteria	3
	β-proteobacteria	31
	γ-proteobacteria	45
Actinobacteria	Corynebacteriales	9

### In silico primer design

To design degenerate primers using HYDEN, the program heuristically receives two important command-line parameters. The initial is an input file containing all the set of DNA sequences of the target genes (i.e, gene under study) in FASTA format, and the latter is a list of command-line parameters specifying the number of primers to design, their length and degeneracy, the regions within the sequences that will be used for designing the primers, the maximal allowable number of mismatches between the primers and the sequences they match, and the parameters, and a number of algorithmic parameters that control the optimization phases of the program when designing primers [7].

**Fig. 2:**
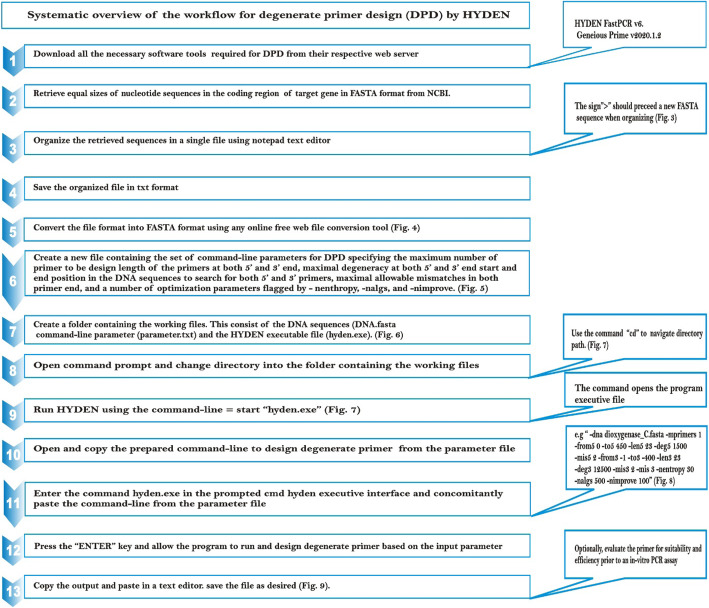
Systematic overview of the workflow for designing degenerate PCR primers via HYDEN heuristics

For the purpose of this study, we have followed the initial method by Linhart and Shamir [7] with a slight modification to design a highly degenerate primer pair targeting bacterial catabolic gene (catA). The design of this primer pair was based on retrieved nucleotide sequences in the coding region of 88 bacterial catabolic genes (catA) encoding the aromatic compound degrading enzyme catechol 1,2-dioxygenase (C12O). To ease the design of the highly degenerate primer pair, a separate file containing the desired command-line parameter was generated prior to running the HYDEN software program. The command-line parameters were thereafter used as input to run the program and design the highly degenerate primer pair. The overall execution of HYDEN using our target genes is illustrated in the workflow given in (Fig. 2).

#### Troubleshooting

It should be noted that the given line “C:\Users\Prosper>” would be exhibited when you open command prompt on any window-based computing system prior to running series of command-lines. The flag “Prosper”, depicts the name of the user of this computer. More also, the command “cd” should be used when changing directories or navigating into different file folders on a window-based computing system prior to the entry of a directory path else, an error message is returned as output. Additionally, directory paths, folder names, or programs name should be entered correctly as written or saved on the computer system. An example of what we meant is described below.

Let us assume that a user of a window-based computer system wants to open a folder named “workspace” containing the hyden executable file located in the Desktop folder of his/her computer in order to design a highly degenerate primer pair, and have entered the command line given below as input;

i.e, Input: C:\Users\Prosper> Desktop workspace hyden

NB: The output of the executed command-line would display the statement given below as output;

Output: 'hyden' is not recognized as an internal or external command, operable program, or batch file.

This had happened because the appropriate command to change the directory path was not used alongside the directory path of interest. It should be noted that the user of this computer system will only be allowed to access the files, scripts, programs, or folder in the specified directory path only when the command “cd” is used to change directories, and the desired folder or directory path is appropriately inputted along with this command.

In order to appropriately open the folder containing the hyden executable file without having an error message as output. The user of this computer system would execute the query as given below;

Input: C:\Users\Prosper> cd Desktop {hit the enter key}

Upon pressing the enter key, this will prompt the line given below;

C:\Users\Prosper\Desktop>

It should be noted that the given command-line above would enable the user to access the files, programs, scripts, or folders contained in the desktop folder;

Once again, the user should use the command “cd” and directory “workspace” to access the hyden executable file as illustrated below;

C:\Users\Prosper\Desktop> cd workspace {hit the enter key}

Upon pressing the enter key, this will prompt the line given below;

C:\Users\Prosper\Desktop\workspace>

**Fig. 3:**
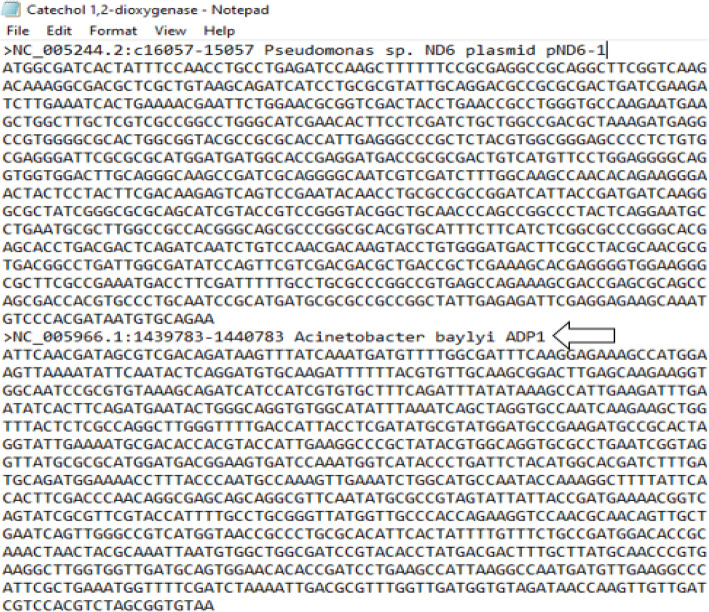
Graphical representation of sequence organization for degenerate primer design. The arrow shows the FASTA identity representation. “>” depicts the FASTA command for a new sequence. NC_005966, 1439783-1440783, *Pseudomonas sp*, ND6 represent the accession number, region of the sequence retrieved, bacterial name, and strain respectively

**Fig. 4:**
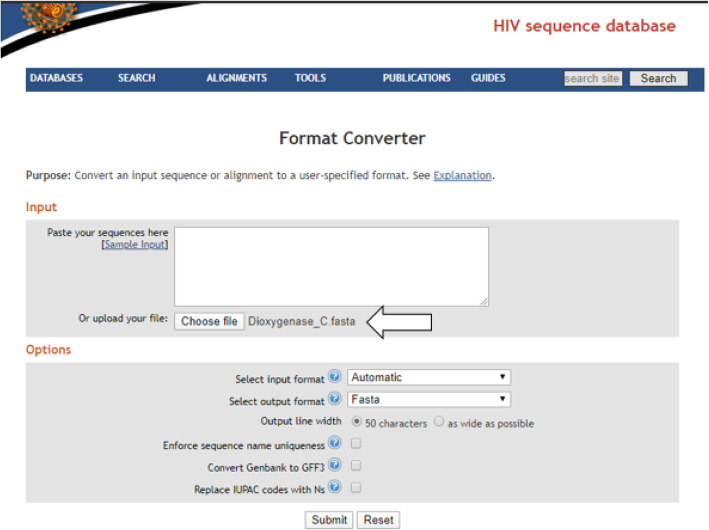
Graphical representation showing the conversion of organized sequences from .txt file extension into fasta sequence format. The arrow shows the prepared sequences organized file

**Fig. 5:**
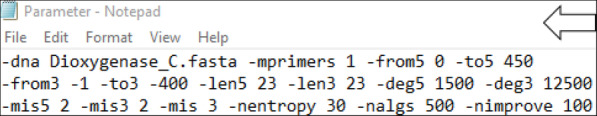
Screenshot of the command-line parameters used to design the degenerate primer pair

NB: The prompted command-line given above allows the user of this computer system to access the files, programs, scripts, or folders in the workspace folder;

Since the hyden executable file is present in this particular folder, the program can therefore be executed afterward (Figs. 3, 4 and 5).

### In silico analysis of primer stats

**Fig. 6:**
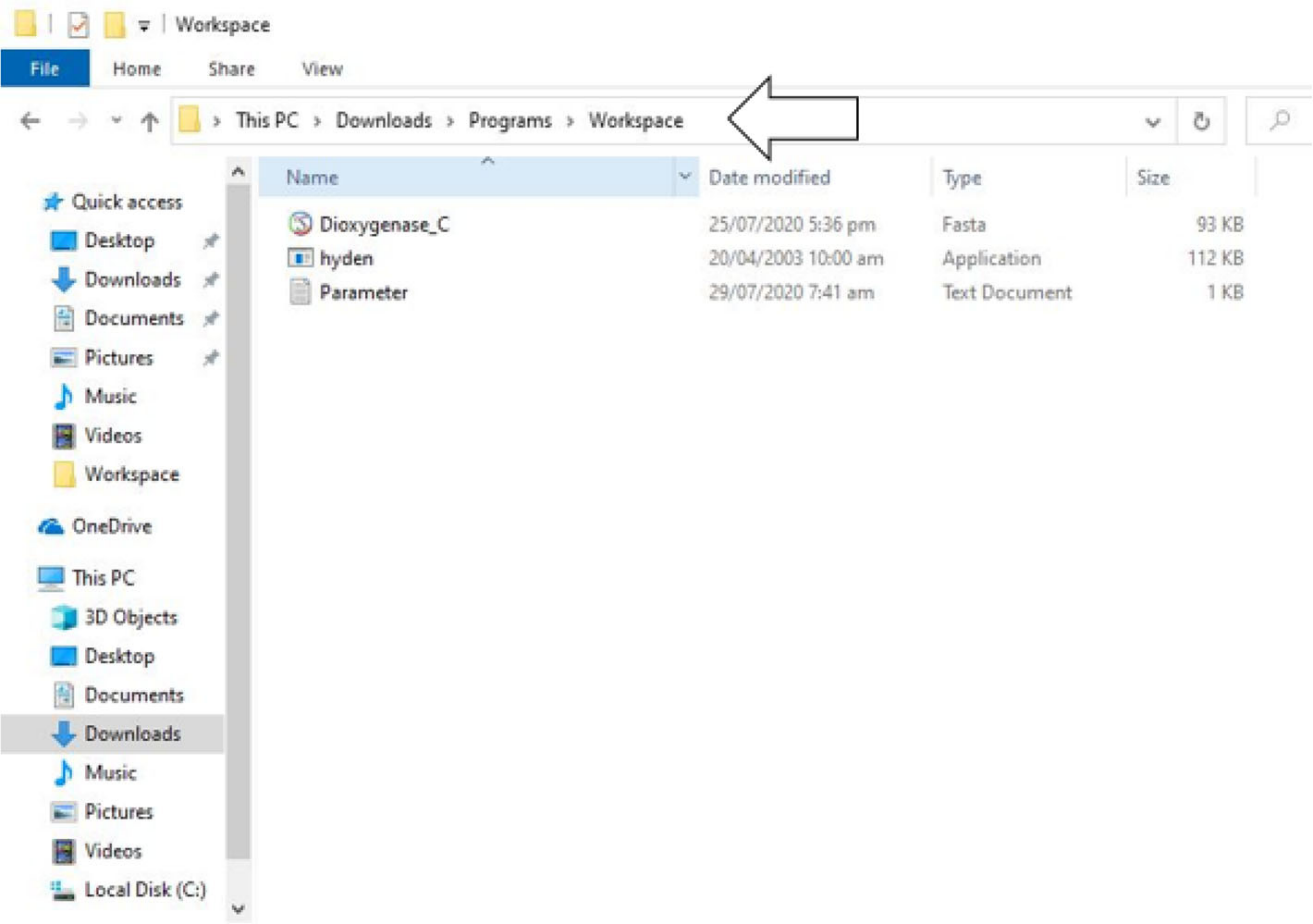
Screenshot illustrating the working folder created for degenerate primer design

**Fig. 7:**
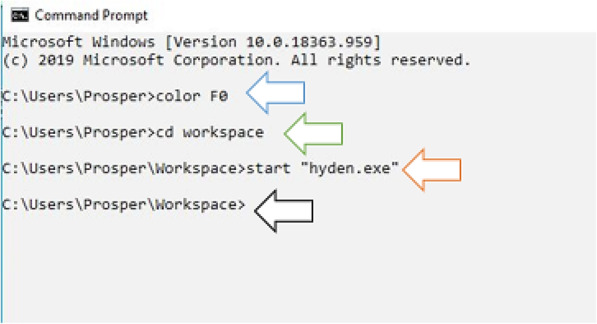
Graphical representation of command actions for degenerate primer design. The arrows show the directory path to the working folder and start-up of the hyden program. The command-line color F0 illustrated by the blue arrow in this figure changes the background of the command prompt as white and the foreground as black. cd workspace changes the directory into the workspace folder as depicted by the green arrow. The orange and black arrow illustrates the opening of the hyden executable file

**Fig. 8:**
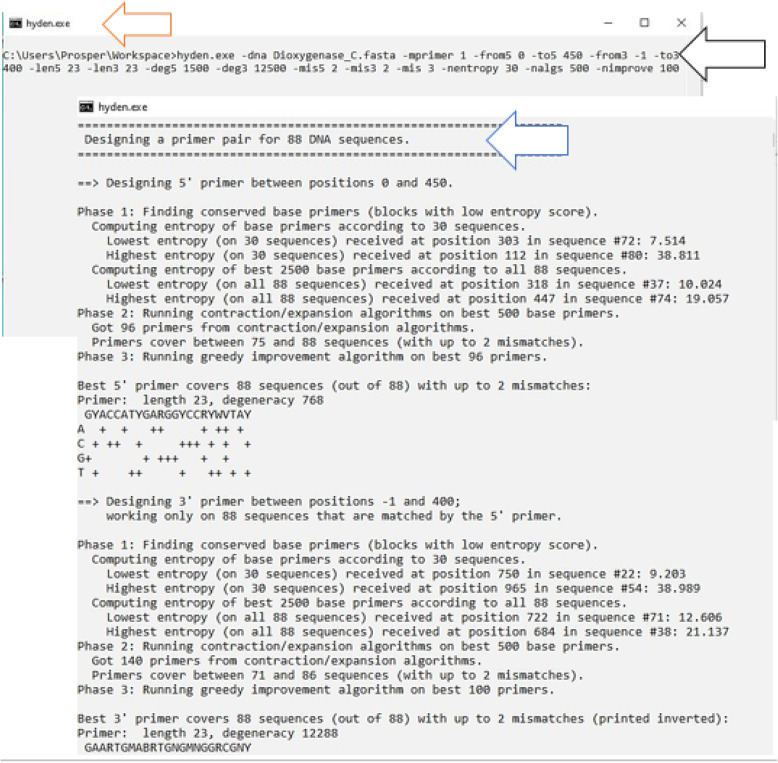
Screenshot illustrating operation of hyden program for degenerate primer design. The orange arrow shows the hyden program interface. The black arrow shows the command-line parameters used in this study for designing the degenerate primer pair. The blue arrow depicts the running of the hyden program for DPD

**Fig. 9:**
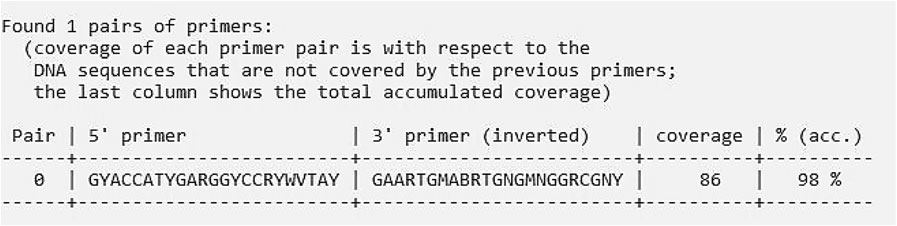
Graphical illustration of hyden-generated output

To analyze the returned primer pair generated by the HYDEN heuristics for sensitivity and suitability towards the target sequences, the primers were evaluated using FastPCR 6.7 program [14]. This program automatically evaluates primers for different properties by calculating their molecular weight, linguistic complexity, and primer PCR efficiency, GC contents, primer extinction coefficient, primer length, unit conversion (nmol per OD), and primer resuspension calculation. The tool is designed to allow the choice of other nearest-neighbor thermodynamic parameters or non-thermodynamic Tm calculation formulae and can also allow users to specify the primer type under evaluation (degenerate or non-degenerate) (Figs. 6, 7, 8 and 9).

### Primer validation via in silico PCR

To confirm the coverage of the newly designed degenerate primer pair on the target sequences and the correspondence with that of the HYDEN generated coverage output (Tables 6, 7, 8, 9, 10, and 11), we tested the designed primer pair on all the 88 sequences reported in this study for coverage via in silico PCR. To simulate the virtual amplification of the genes with our designed primer pair, in silico PCR was performed using Geneious Prime software (version2020.1.2) [15]. To run the program, all target sequences were imported as separate sequences and were screened for primer binding positions, allowing for up to 3 mismatches in the primer binding region and 2 mismatches at the 3’ end of the primer.

## Results

### Retrieval of sequence

A total of 88 nucleotide sequences (all of 1 kb in size each in the coding region) of catA genes from different bacteria species were retrieved from the NCBI database and were used in this study to design a highly degenerate primer pair using the HYDEN software program.

### Primer designed by HYDEN

The results of the designed primer pair by the heuristic program of choice are summarized in (Table 2). The last column indicates the number of (i.e, percentage) of genes (out of 88 sequences) that is matched (i.e, covered) by the primer pair with up to 3 mismatches in both primer ends combined. It can be emphasized that the primer pair (catAf and catAr) binds to 87 sequences out of the 88 catA sequences that were used in designing the degenerate primer pair with a total accumulated percentage of (98%) of the genes. Although all the selected bacterial strains used in this study to design the highly degenerate primer pair possess the C12O genes, nonetheless, the failure of primer catAf and catAr to bind the residual gene sequence might conceivably result from lower homology between the sequences of the gene and that of the C12O primer pair.

**Table 2:** Degenerate primer pairs for amplification of aromatic compound dioxygenase gene families.

Primers	Sequence^a^ (5’–3’)	Degeneracy^b^	Coverage^c^	%^d^ (acc.)
catAf	GYACCATYGARGGYCCRYWVTAY	768	87	98
catAr	GAARTGMABRTGNGMNGGRCGNY	12288		

^a^All the oligonucleotide primers listed in the table above are in 5’–3’ direction. Nucleotide bases other than the standard Watson-crick bases depict the wobble bases. *M* (A/C), *R* (A/G), *W* (A/T), *Y* (C/T), *S* (C/G), *V* (A/C/G), *B* (C/G/T), *N* (A/C/G/T). It is noteworthy that all the designed primer pairs are non-degenerate at the 5’ end which serves as a consensus clam region and are degenerated at the 3’ end. ^b^is the total degeneracy present in individual primers. The program introduces degeneracies at some positions within the primers so that they would amplify a maximum number of the input sequences as well as other novel genes within the gene superfamily. ^c^This specifies the number of genes (out of the 160) sequence sets that each primer matches. ^d^The last column is the total accumulation coverage. It depicts the percentage of the input sequences covered by the combinations of all three primers

### In silico evaluations of primer property

(Tables 3, 4, 5). Contains all the primer parameters calculated by applying FastPCR v6.7 software program (http://primerdigital.com/Fastpcr.html) [14]. The program calculates the primer melting temperature using default or other formulae for both normal and degenerate sequence combinations, G-C content, molar extinction coefficient, unit conversion (nmol per OD), mass (μg per OD), molecular weight, linguistic complexity, primer PCR efficiency [16], and also allow the predictions of several thermodynamic parameters and secondary non-specific binding. It is noteworthy that in a real in vitro PCR reaction, several primer properties are of pertinence to ensure a successful amplification more of which the primer length, % GC, primer efficiency, and melting temperature (Tm) are key dictates.

**Table 3:** Degenerate PCR primer properties

Primers	%GC	A	G	T	C	μg/OD_260_	L.C (%)	PPe (%)	∆G kcal/mol
catAf	52.9	5.8	5.3	5.0	6.8	31.722	88	87	−29.0
catAr	57.2	6.3	9.6	3.6	3.6	30.985	95	51	−31.1

*F* Forward, *R* Reverse, *GC* Guanine-cytosine percentage, *AGTC* Number of adenine, guanine, thymine, and cytosine residues present in primer, *OD* Optical density, *LC* Linguistic complexity, *PPe* Primer’s PCR efficiency, *∆G* Change in free energy

**Table 4:** Degenerate PCR primer properties

Primers	M.W (g/mol)	∆H (kcal/mol)	∆S (cal/kmol)	Tm (°C)	Tm(ATP) (°C)	ε L/(mol.cm)	nmol/OD_260_
catAf	7018	−175.4	−496.8	58.0	60.6	221233	4.520
catAr	7177	−180.7	−506.9	61.6	62.4	231625	4.317

*MW* Molecular weight, *∆H* Enthalpy change, *∆S* Entropy change, *Tm* Melting temperature, *ATP* Allawi's thermodynamics parameters, *ε* Extinction coefficient, *nmol/OD*_*260*_ Unit conversion

**Table 5:** Degenerate primer evaluation for secondary structure

Primers	Predicted secondary structure	Tm (°C)	∆G kcal/mol
catAf	**–**	**–**	**–**
catAr	Internal or 5'end dimer	36.8	−15.0

*Tm* Melting temperature, *∆G* Change in free energy

Therefore, it is essential that these parameters be screened before a primer should be used in an actual in vitro PCR assay to minimize or eliminate the likeliness for PCR failure. Primers are termed good when they strike an equilibrium between stringency, coverage, and efficiency. Notably, primer binding in the intended region of DNA sequences is primarily controlled by the length and annealing temperature (Ta) [3]. Interestingly, the flexibility of HYDEN to enable users to design primers of the desired length is advantageous to maintain constancy of primer sizes. The result of the primer statistics from this study revealed an equal primer length of 23 bp in size. Since primers are best between 18 and 24 bp [3] for improved specificity, the likeliness that our designed primers would correctly bind to target sequences may exist.

More importantly, the amount of guanine and cytosine bases contained within a primer is of pertinence when considering its success in in-vitro PCR studies. The combination of these two bases dictates both the melting temperature (Tm) and the annealing temperature (Ta) which ultimately defines the primer binding mode to target sequences. Considerably, a guanine-cytosine percentage between 40 and 60% is generally reported for a good PCR reaction. Nonetheless, this may vary depending on the type of primer, as well as the kind of PCR approach under consideration. It is well known that higher % GC more often result in non-specific annealing. From the result of our analysis with the designed primer pair, it can be emphasized that primer catAf and catAr satisfactorily met the % GC constraint with a GC content of 52.9% and 57.2% respectively amounting to a melting temperature of 58.0 °C and 61.6 °C respectively which can be considered appropriate for PCR reaction since it is within the range of the generally accepted melting temperature limits that range from 56–62 °C.

Additionally, the primer pair showed a significant level of PCR efficiency. The analysis revealed catAf to have an efficiency of 87% and catAr to exhibit 51% efficiency. For a highly degenerate primer, the 5’ end generally depicts the non-degenerate consensus clamp while the 3’ end is the degenerated region. It is a well-established fact that higher degeneracy would likely affect primer efficiency. Thus, the low efficiency of the reversed primer catAr may be correlated to elevated degeneracy at specific positions of the oligonucleotide. In amplification studies, primer efficiency often presents the rationale for the success of a PCR reaction and is more commonly helpful to rationalize the amplicon size generated in the PCR reaction.

As earlier discussed FastPCR v6.7 software program (http://primerdigital.com/Fastpcr.html) [14] could also allow the evaluation of primers for possible secondary structure. Sequence homology between primer pairs or within primers may affect primer-template annealing which in most times would negatively affect sequence amplification downstream. While screening our designed primer pair, an internal secondary structure was predicted by FastPCR software program with primer catAr. From our result, the possibility for dimer structure at the 5’ end of primer catAr was found to occur at 36.8 °C with a ∆G value of −15.0 Kcal/mol (Table 5). Generally, more negative free energy changes below −6 Kcal/mol would most likely affect the product yield. More also, this could also affect the primer-template binding by reducing the availability of primers to the reaction. Nonetheless, the failure for secondary interactions that could result in cross dimerism (heterodimer formation) between the sequences of catAf and catAr is quite promising.

Furthermore, the temperature at which secondary structures are formed with primers is another factor to consider when designing any primer since they ultimately affect the general PCR reaction by either interfering with yield or resulting in the overall PCR failure. More interestingly, primer catAr formed an internal structure at a temperature below the regular PCR temperature cycle. Putatively, this would result in little or no effect on the amplification product and may be neglected. Owing to the evidences from this analysis, we emphasize that the designed primers fit in the design constraint and could conceivably amount to a reasonable level of success in PCR reactions.

### In silico PCR validation of designed primer with input sequences

**Fig. 10:**
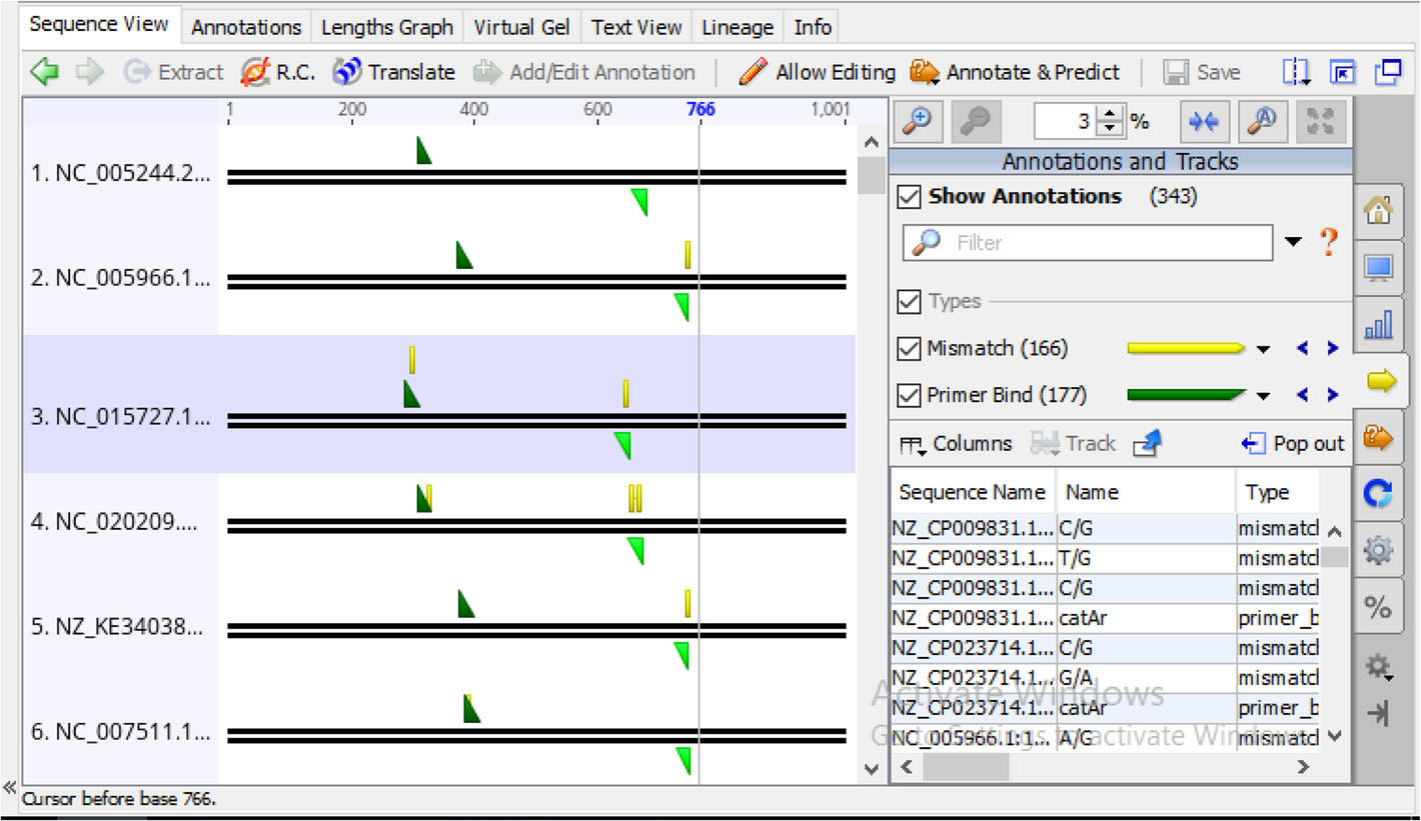
In silico PCR result showing amplification of catechol 1,2-dioxygenases (C12O) gene with HYDEN designed degenerate primer catAf and catAr. The thick dark parallel non-overlapping lines in the figure represent both strands (Sense and antisense) of DNA in the orientation 5’-3’ and 3’-5’ respectively. The right-angle triangles depict the primers. Green right-angle triangles indicate primer-template binding in the 5’region of the sense strand while inverted olive green right-angle triangle illustrates primer-template binding in the 5’region of the antisense strand. All yellow figures depict mismatches between sequences in the primers and target

Several computer algorithms have been written to authenticate PCR primers, many of which allow the simulation of PCR reaction with designed primers to tests for specificity, efficiency, and coverage with target sequences before an actual in vitro PCR assay. These programs enables the prediction of amplicons from either a sequence database or a set of sequence inputs. The technique of using any computer-based program that relies on one or more theoretical heuristics or computational algorithms to predict or calculate the outcome of a PCR amplification product is generally referred to as in silico PCR. In this study, our newly designed degenerate primer pair catAf and catAr were tested in silico for their efficacy to bind to the bacteria catechol 1,2-dioxygenases (catA) gene and for their ability to maintain corresponding coverages with that of the HYDEN generated coverage output obtained subsequent to the degenerate primer design via its heuristics. The result of the in silico PCR amplification is summarized in (Tables 6, 7, 8, 9, 10, and 11). Comparatively, the result from the in silico PCR validation study, showed an approximately equal number of sequence coverage with that of the earlier number of coverages pre-defined by the HYDEN software program. Similar to the HYDEN output, primer catAf and catAr cohesively covered all the sequences used for the degenerate primer design allowing a total number of 166 mismatches with the target sequences (Fig. 10).

**Table 6:** In silico PCR analysis of primer pair catAf/catAr with targeted sequences

Bacterial strains	Accession numbers	Product size	Product length	Primer dimer Tm (°C)
*Pseudomonas sp.* ND6	NC_005244.2	371	308–678	24.0
*Acinetobacter baylyi* ADP1	NC_005966.1	374	373–746	24.0
*Cupriavidus necator* N-1	NC_015727.1	366	286–651	24.0
*Pseudomonas poae* RE*1-1-14	NC_020209.1	365	308–672	24.0
*Acinetobacter colistiniresistens* NIPH 2036	NZ_KE340381.1	371	376–746	24.0
*Burkholderia lata*	NC_007511.1	365	385–749	24.0
*Pseudomonas* fulva 12-X	NC_015556.1	365	379–743	24.0
*Pseudomonas stutzeri*	NC_015740.1	371	370–740	24.0
*Ralstonia mannitolilytica* SN82F48	NZ_CP010799.1	374	308–681	24.0
*Burkholderia pseudomultivorans* SUB-INT23-BP2	NZ_CP013377.1	374	373–746	24.0
*Pseudomonas chlororaphis* subsp. *aurantiaca* strain DSM 19603	NZ_CP027746.1	365	308–672	24.0
*Burkholderia stagnalis* MSMB735WGS	NZ_CP013461.1	365	287–651	24.0
*Burkholderia territorii* strain RF8-non-BP5	NZ_CP013365.1	365	287–651	24.0
*Burkholderia anthina* strain AZ-4-2-10-S1-D7	NZ_LNJP01000003.1	365	385–651	24.0
*Burkholderia contaminans* MS14	NZ_CP009745.1	374	317–690	24.0
*Pseudomonas savastanoi pv. savastanoi* NCPPB 3335	NZ_CP008742.1	365	403-767	24.0

**Table 7:** In silico PCR analysis of primer pair catAf/catAr with targeted sequences

Bacterial strains	Accession numbers	Product size	Product length	Primer dimer Tm (°C)
*Burkholderia mallei* ATCC 23344	NC_006349.2	365	287–651	24.0
*Pseudomonas syringae* pv. *actinidiae* str. Shaanxi_M228	NZ_CP032631.1	365	308–672	24.0
*Burkholderia cenocepacia* J2315	NC_011001.1	365	287–651	24.0
*Burkholderia pseudomallei* MSHR2543	NZ_CP009477.1	365	385–749	24.0
*Burkholderia glumae* LMG 2196	NZ_CP009434.1	374	311–684	24.0
*Burkholderia ambifaria* AMMD	NC_008391.1	365	287–651	24.0
*Paraburkholderia hospita* DSM 17164	NZ_CP026106.1	374	308–681	24.0
*Pseudomonas avellanae* R2leaf	NZ_CP026562.1	365	403–767	24.0
*Oligella urethralis* DSM 7531	NZ_AQVB01000020.1	374	308–681	24.0
*Acinetobacter soli* GFJ2	NZ_CP016896.1	374	308–681	24.0
*Burkholderia cepacia* ATCC 25416 strain UCB 717	NZ_CP012983.1	374	317–690	24.0
*Acinetobacter bereziniae* XH901	NZ_CP018259.1	371	296–666	24.0
*Pseudomonas mandelii* LMG 21607	NZ_LT629796.1	365	308–678	24.0
*Bordetella trematum* H044680328	NZ_LT546645.1	374	308–678	24.0

**Table 8:** In silico PCR analysis of primer pair catAf/catAr with targeted sequences

Bacterial strains	Accession numbers	Product size	Product length	Primer dimer Tm (°C)
*Ensifer adhaerens* Casida A	NZ_CP015881.1	374	308-681	24.0
*Pluralibacter gergoviae* FB2	NZ_CP009450.1	371	296-666	24.0
*Burkholderia gladioli* ATCC 10248	NZ_CP009322.1	365	293-657	24.0
*Klebsiella quasipneumoniae* strain ATCC 700603 isolate K6	NZ_CP014696.2	365	308-672	24.0
*Pseudomonas putida* NBRC 14164	NC_021505.1	365	308-672	24.0
*Acinetobacter venetianus* VE-C3	NZ_CM001772.1	371	296-666	24.0
*Pseudomonas monteilii* B5	NZ_CP022562.1	365	308-672	24.0
*Burkholderia lata*	NC_007509.1	374	317-690	24.0
*Acinetobacter indicus* CIP 110367	NZ_KI530745.1	371	296-666	24.0
*Acinetobacter junii* 65	NZ_CP019041.1	371	296-666	24.0
*Acinetobacter johnsonii* XBB1	NZ_CP010350.1	371	296-666	24.0
*Pseudomonas protegens* CHA0	NC_021237.1	365	308-672	24.0
*Cupriavidus metallidurans* CH34	NC_007974.2	371	299-669	24.0
*Acinetobacter* *radioresistens* DSM 6976	NZ_KB849749.1	371	296-666	24.0
*Pseudomonas aeruginosa* PAO1	NC_002516.2	365	308-672	24.0
*Sphingomonas* sp. KA1	NC_008308.1	371	302-672	24.0

**Table 9:** In silico PCR analysis of primer pair catAf/catAr with targeted sequences

Bacterial strains	Accession numbers	Product size	Product length	Primer dimer Tm (°C)
*Pseudomonas aeruginosa* DSM 50071	NZ_CP012001.1	365	308-672	24.0
*Cupriavidus necator* N-1	NC_015726.1	371	299-669	24.0
*Burkholderia ubonensis* MSMB22	NZ_CP009486.1	371	296-666	24.0
*Burkholderia anthina* strain AZ-4-2-10-S1-D7	NZ_CM003769.1	374	323-696	24.0
*Pseudomonas azotoformans* S4	NZ_CP014546.1	371	308-672	24.0
*Pseudomonas lactis* strain DSM 29167	NZ_JYLO01000007.1	365	379-743	24.0
*Pseudomonas simiae* PCL1751	NZ_CP010896.1	365	308-672	24.0
*Acinetobacter johnsonii* XBB1	NZ_CP010351.1	371	296-666	24.0
*Acinetobacter pittii* PHEA-2	NC_016603.1	371	296-666	24.0
*Rhodococcus qingshengii* djl-6-2	NZ_CP025959.1	371	299-666	24.0
*Rhodococcus opacus* B4	NC_012522.1	371	299-669	24.0
*Pseudomonas brassicacearum* subsp. *brassicacearum*	NC_015379.1	365	308-672	24.0
NFM421 *Burkholderia thailandensis* E264	NC_007650.1	365	287-651	24.0
*Burkholderia cenocepacia* J2315	NC_011002.1	374	314-687	24.0

**Table 10:** In silico PCR analysis of primer pair catAf/catAr with targeted sequences

Bacterial strains	Accession numbers	Product size	Product length	Primer dimer Tm (°C)
*Burkholderia multivorans* ATCC BAA-247	NZ_CP009831.1	365	287-651	24.0
*Bordetella holmesii* ATCC 51541	NZ_CP007494.1	374	308-681	24.0
*Rhodococcus pyridinivorans* SB3094	NC_023150.1	371	305-675	24.0
*Rhodococcus rhodochrous* NCTC10210	NZ_LT906450.1	371	305-675	24.0
*Burkholderia cepacia* ATCC 25416 strain UCB 717	NZ_CP012982.1	365	287-651	24.0
*Burkholderia vietnamiensis* LMG 10929	NZ_CP009630.1	365	385-749	24.0
*Pseudomonas proteolytica* LMG 22710	NZ_FNTR01000004.1	365	308-672	24.0
*Pseudomonas fluorescens* NCTC10038	NZ_LS483372.1	365	308-672	24.0
*Sinorhizobium fredii* CCBAU 25509	NZ_CP029451.1	374	308-681	24.0
*Pseudomonas veronii* strain R02	NZ_CP018420.1	365	308-672	24.0
*Klebsiella grimontii* SS141	NZ_CP044527.1	365	308-672	24.0
*Pseudomonas marginalis* ICMP 3553 scaffold44	NZ_LKEG01000057.1	365	379-743	24.0
*Pseudomonas plecoglossicida* XSDHY-P	NZ_CP031146.1	365	308-672	24.0

**Table 11:** In silico PCR analysis of primer pair catAf/catAr with targeted sequences

Bacterial strains	Accession numbers	Product size	Product length	Primer dimer Tm (°C)
*Burkholderia ambifaria* AMMD	NC_008392.1	374	317-690	24.0
*Rhodococcus ruber* YC-YT1	NZ_CP023714.1	371	451-821	24.
*Mycolicibacterium smegmatis* NCTC8159	NZ_LN831039.1	365	353-717	24.0
*Rhodococcus erythropolis* BG43	NZ_CP011295.1	368	299-666	24.0
*Burkholderia vietnamiensis* LMG 10929	NZ_CP009632.1	374	373-746	24.0
*Klebsiella aerogenes* KCTC 2190	NC_015663.1	365	308-672	24.0
*Acinetobacter seifertii* NIPH 973	NZ_KB851200.1	371	296-666	24.0
*Escherichia fergusonii* ATCC 35469	NC_011740.1	365	308-672	24.0
*Rhodococcus fascians* D188	NZ_CP015235.1	368	329-696	24.0
*Kocuria rosea* ATCC 186	NZ_CP035103.1	368	326-693	24.0
*Acinetobacter schindleri* SGAir0122	NZ_CP025618.2	371	296-666	24.0
*Acinetobacter nosocomialis* strain 6411	NZ_CP010368.1	371	296-666	24.0
*Pseudomonas fragi* NMC25	NZ_CP021132.1	365	308-675	24.0
*Acinetobacter calcoaceticus* CA16	NZ_CP020000.1	371	296-666	24.0
*Acinetobacter oleivorans* DR1	NC_014259.1	371	296-666	24.0

Furthermore, the results of the simulations indicated a constancy in amplicon size. The primers were also observed to produce products ranging from 365–374 bp with a binding pattern spanning across position 287–821 of the target sequences (Tables 6, 7, 8, 9, 10, and 11). The amplicon sizes produced, also corresponded with an early degenerate primer set raised by Sei et al. [17] that could effectively amplify catechol 1,2-dioxygenase genes present in bacteria giving rise to an amplicon size of approximately 282 bp and was found to amplify a fragment of C12O gene in *Pseudomonas putida* N6 resulting in an amplicon size of 350 bp [18]. Noteworthy, virtual PCR tests with our designed primer pair on the target sequences amounted to a total of zero off target in all binding mode indicating a high specificity with the gene targets. Presumably, our newly designed degenerate primer pair (catAf and catAr) may possibly amplify specific fragments from a wide variety of catechol 1,2-dioxygenase (C12O) genes in other referenced bacterial strains that possess this catabolic gene.

### In silico PCR validation of primer *catA* using referenced bacterial strains

**Fig. 11:**
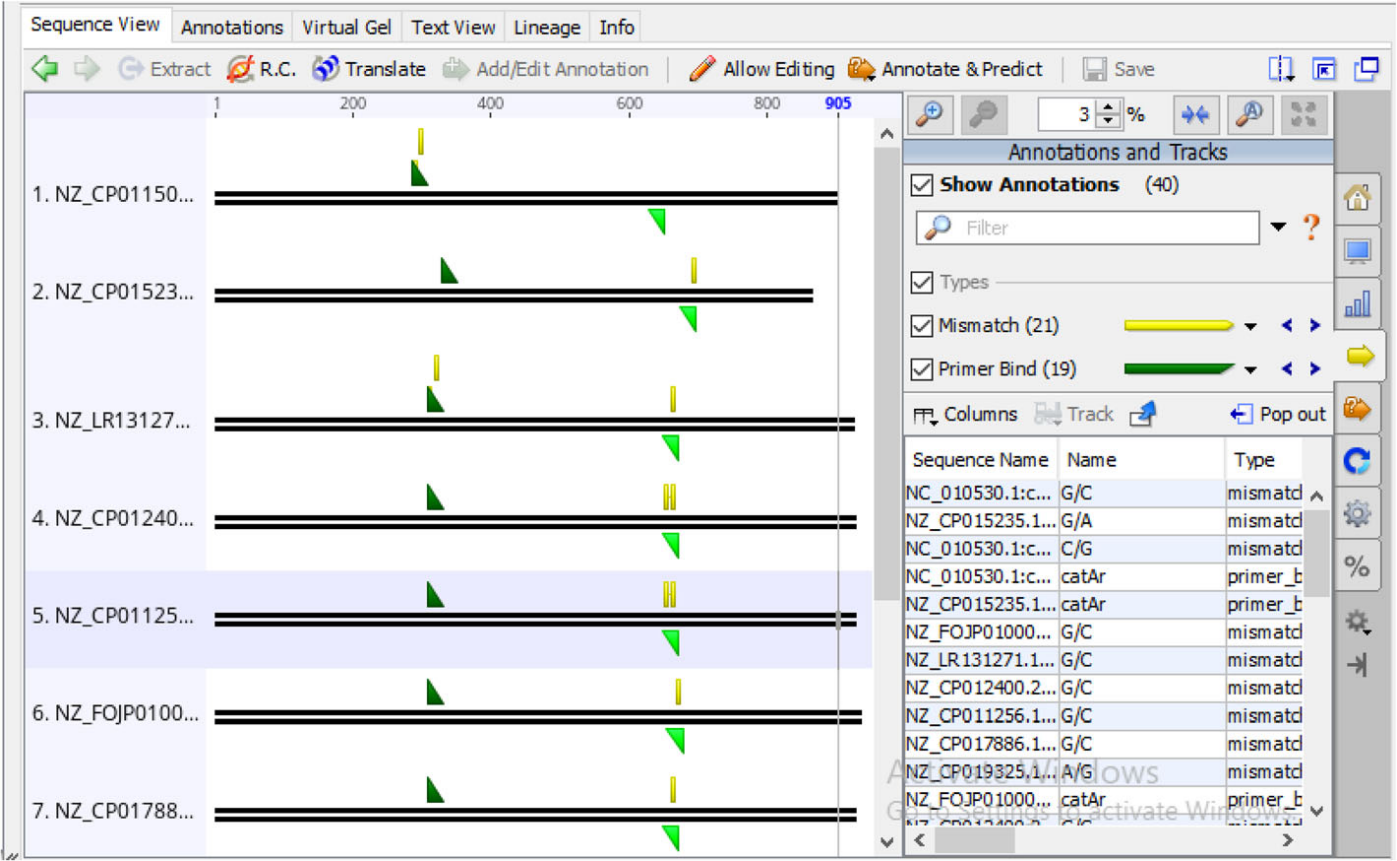
In silico PCR result showing amplification of catechol 1,2-dioxygenase (C12O) gene fragments in authentic bacterial strains with C12O genes

**Fig. 12:**
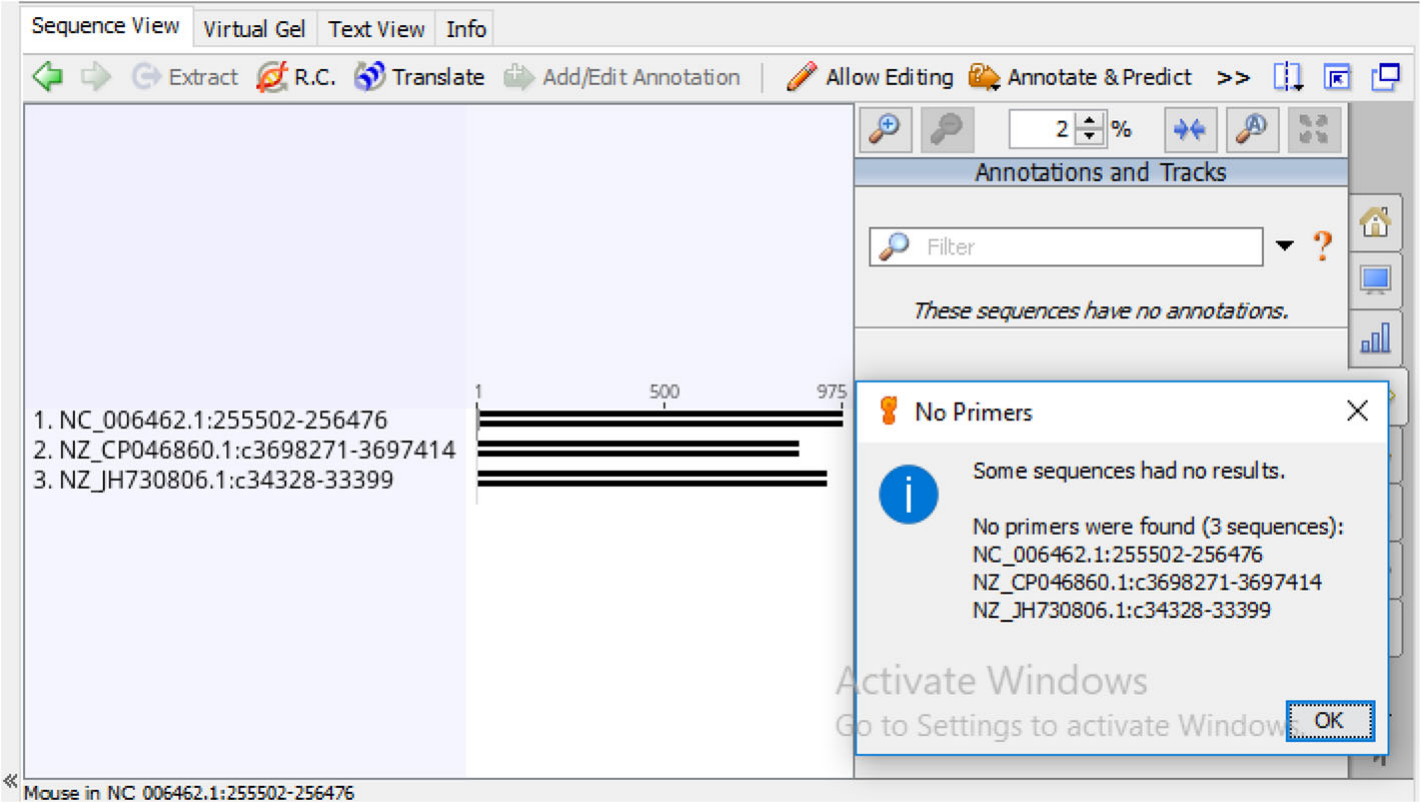
In silico PCR result of authentic bacterial strains without catechol 1,2-dioxygenase (C12O) genes

To confirm the likeliness for extensive amplification of C12O genes and to test the specificity of the newly designed primer catAf and catAr, we re-conducted another validation study through in silico PCR on 10 authentic bacterial strains known to carry the C12O genes and are equally not used for the primer design (Table 12). Additionally, 3 bacterial strains without C120 genes were also used as negative controls for specificity [17] (Table 13). The result of our demonstration revealed that the primer pair could correspondingly amplify the 10 sequences producing a product size ranging from 365–374 bp in the same manner as those of the sequences used for the primer design (Fig. 11). However, primer catAf was unable to find sequence matches with *Cupriavidus taiwanensis* LMG 19424 which have been thought to result from low sequence complementarity between its sequences and the template. More also, a total of 21 mismatches was observed from the virtual amplification. Furthermore, no fragment was amplified from the negative controls (Fig. 12).

**Table 12:** In silico PCR result of primer pair catAf/catAr with authentic bacterial strains with (positive control) catechol 1,2-dioxygenase (C12O) gene

No.	Bacterial strains	Accession numbers	Amplification results
			*catA*
1	*Burkholderia pyrrocinia* DSM 10685	NZ_CP011504.1	**+**
2	*Raoultella terrigena* NCTC13098	NZ_LR131271.1	**+**
3	*Pseudomonas yamanorum* LBUM636	NZ_CP012400.2	**+**
4	*Xanthomonas arboricola* 17	NZ_CP011256.1	**+**
5	*Pseudomonas otitidis* DSM 17224	NZ_FOJP01000011.1	**+**
6	*Pseudomonas frederiksbergensis* strain ERDD5	NZ_CP017886.1	**+**
7	*Klebsiella quasivariicola* KPN1705	NZ_CP022823.1	**+**
8	*Cupriavidus taiwanensis* LMG 19424	NC_010530.1	**±**
9	*Achromobacter insolitus* DSM 23807	NZ_CP019325.1	**+**
10	*Rhodococcus fascians* D188	NZ_CP015235.1	**+**

+ Amplification, **±** Partially amplified

**Table 13:** In silico PCR result of primer pair catAf/catAr with known bacterial strains without (negative control) catechol 1,2-dioxygenase (C12O) gene

No.	Bacterial strains	Accession numbers	Amplification results
			*catA*
1	*Thermus thermophilus* HB8	NC_006462.1	–
2	*Pseudomonas gingeri* NCPPB 3146	NZ_JH730806.1	–
3	*Bacillus subtilis* RS10	NZ_CP046860.1	–

– No amplification

## Discussion

The majority of the computational tools developed to effectively and efficiently address the DPD problem while simultaneously managing high-throughput sequences are extensively run by the command line. However, the lack of a graphical user interface to simplify access to many of these tools could be quite challenging for non-bioinformatics users or researchers with relatively little or no knowledge in computer-assisted programs since many of these tools often require an appreciable level of expertise to effect use. In spite of this constrain, many of these programs still holds a very good profile to design and optimize degenerate primers while fitting in the DPD constraint. For example, the highly degenerate primer design program HYDEN [7] an earlier algorithm based on a heuristic approach primarily addresses a variant of the DPD problem termed maximum coverage degenerate primer design (MC-DPD) which attempts to find a primer of length *l* and degeneracy at most *d*_max_ that covers a maximum number of sequences of a given input set, each of a definite length *l* [13].

Prior to the development of the HYDEN program, finding primers that could extensively cover a broad range of input sequences, and simultaneously striking a balance between coverage and degeneracy was the main concern. Though degeneracy had earlier played a vital role in degenerate primer design allowing these oligonucleotides to cover a large number of known genes and was also thought to provide the chances to detect newer related ones. Nonetheless, higher degeneracy in primers could increase the probability of amplifying unrelated sequences thus, decreasing specificity [13].

To date, several programs enabling degenerate primer design has been proposed and developed to solve the primer accessibility constraints through a graphical user interface. For example, CODEHOP [8] and DePiCt [9] both allow the design of degenerate primers from aligned protein sequences to identify new members of protein families through a GUI. However, their inability to construct primers with high degeneracy on large sets of long genomic sequences could render them inappropriate for larger input strings [13]. Although a good number of the newer DPD programs that could provide users with a GUI such as DegePrime [12] and FAS-DPD [5] could also design primers based on very large sequence size at an order of several thousands to millions, nonetheless, they mechanistically address the MC-DPD problem based on HYDENs heuristics with only a few computational modifications made.

To date, HYDEN remains a notable DPD tool. It heuristics present an ultimate premium for designing several degenerate primers that could be used to detect and find newer genes within a protein superfamily and could sufficiently do this within a few computational time.

To design degenerate primers, HYDEN does this by running a three-phase algorithm. In an attempt to select appropriate primers that would fit into the length and degeneracy constraints, the program first scores sub-sequences appearing in the input DNA sequences from which the primer candidates are chosen and extracted. In the second phase, these candidates are subjected to a simple approximation algorithm called H-CONTRACTION and H-EXPANSION. In the contraction procedure, HYDEN iteratively screens fully degenerate primer candidates and mechanistically discard characters at degenerated sites with the smallest degeneracy count until the primers reach the required degeneracy [7]. Accordingly, non-degenerate candidates are also iteratively expanded to degenerate primers by introducing bases with very large degeneracy count until a threshold is attained. At the final phase, HYDEN attempt to improve these primers by employing a simple hill-climbing procedure, called H-GREEDY which meticulously screens the primers for the possibility of substituting nucleotides in degenerated sites so as to increase primer coverage.

Fortunately, the output generated by the program also provides users with the option to make quick and easy changes to the designed primers or input sequences due to its computational flexibility. In spite of these benefits, there is still a need to simplify access to using this tool for designing degenerate primers via comprehensive approaches. To the best of our knowledge, there has been no study until now to simplify access to using this primer design program. Knowing this, we have attempted in this study to provide a systematic and a user-friendly approach to design degenerate primers using the HYDEN software heuristics.

The results from the in silico assessment performed by two validation tools (FastPCR v6.7 and Geneious prime version2020.1.2) for primer evaluations and product prediction substantiated the correspondence between efficiency, coverage, and the specificity of our designed primer pair to extend the target sequences as pre-defined by the HYDEN software heuristics, thus validating the fascinating potentials of the program.

## Conclusion

In this current study, we demonstrated a systematic approach for degenerate primer designing and evaluation methods that could accurately reduce the chances of synthesizing and optimizing false positive or decoy degenerate PCR primers. The current study also addresses the accessibility constraint of designing highly degenerate primers through programs that are extensively run by command-line which have been thought to be the main problem decreasing the number of degenerate primers more recently reported since many of these reliable degenerate primer design programs often lacks Graphical User Interface (GUI) to simplify use in spite holding a good profile to design degenerate primers.

To make this study more specific, we selected the Highly Degenerate primer (HYDEN) design program as our program of choice owing to the observed reduction in the number of users designing and selecting degenerate PCR primers with its heuristics from literature search compared with similar degenerate primer design programs having the same accessibility constraints. For the purpose of this study, we have designed a highly degenerate primer pair targeting a set of catechol 1,2-dioxygenase (C12O) genes among 88 bacterial strains using the heuristics of HYDENs program. This present study would enable non-bioinformaticians or researchers with relatively little or no knowledge in computer-aided programs to self-design highly degenerate primers of their choice that could be channeled for use in various research study of similar interest rather than relying on previously designed or reported degenerate primers which might not be suitable for use in their respective research.

Morealso, we also believed that we might have concomitantly designed a promising primer candidate that might extensively amplify specific fragments of catechol l,2-dioxygenase (C120) gene from a wide variety of bacterial populations owing to the success of the designed degenerate primer pair in in-silico PCR validation studies. However, there would be a need for an actual in-vitro PCR assay of our designed degenerate primer pair to further validate its suitability for detecting the bacterial catabolic gene C12O before its consideration for use.

## Data Availability

We declare that all the data generated are included in this study.

## Abbreviations

DPD: Degenerate primer design
HYDEN: Highly Degenerate
MC-DPD: Maximum coverage-degenerate primer design
PCR: Polymerase chain reaction
GUI: Graphical user interface
DNA: Deoxyribose nucleic acid
GC: Guanine cytosine
CODEHOP: Consensus-degenerate hybrid oligonucleotide primer
FAS-DPD: Family-specific degenerate primer design
DPP: Degenerate PCR primer
NCBI: National Center for Biotechnology Information
C12O: Catechol 1,2-dioxygenase
OD: Optical density
Tm: Melting temperature
Ta: Annealing temperature
d_max_: Maximum degeneracy
L: Length
-dna: DNA file in fasta format
-mprimers: Maximal number of primer pairs to design
-len5: Length of 5' primer
-deg5: Maximal degeneracy of 5' primer
-mis5: Maximal number of mismatches in 5' end
-len3: Length of 3' primer
-deg3: Maximal degeneracy of 3' primer
-mis3: Maximal number of mismatches in 3' end
-nentropy: number of DNA sequences for entropy estimation
-mis: Maximal number of mismatches in both ends combined
nalgs: number of base primers to run contraction/expansion algorithms
-nimprove: number of best candidates to run greedy improvement algorithm
exe: executable
txt: text
cmd: command
cd: change directory
